# Portable OCT-assisted surgical treatment of intracorneal pre-Descemet epithelial cyst: a case report

**DOI:** 10.1186/s12886-017-0558-4

**Published:** 2017-08-29

**Authors:** Sang Woo Kim, Eung Kweon Kim

**Affiliations:** 1 Department of Opthalmology, Ulsan University Hospital, Ulsan University of College of Medicine, Ulsan, South Korea; 2 Department of Ophthalmology, Corneal Dystrophy Research Institute, Institute of Vision Research, Severance Biomedical Science Institute, Yonsei University College of Medicine, 134 Shinchon-dong, Seodaemun-ku, Seoul, 120-752 South Korea

**Keywords:** Case report, Deep-seated intracorneal epithelial cyst, Fourier-domain optical coherence tomography, 10% Trichloroacetic acid

## Abstract

**Background:**

Intracorneal epithelial cysts are a rare clinical condition that can occur anywhere in the corneal tissue; however, they appear most commonly in the stroma. They are sometimes challenging to treat because of their location, depth, and visual outcomes. Herein, we report a pre-Descemet epithelial cyst that was successfully treated surgically, with guidance from Fourier-domain optical coherence tomography (FD-OCT).

**Case presentation:**

This interventional case report presents a patient with gradually decreasing vision caused by a pre-Descemet epithelial cyst. A 4-year-old girl with no history of trauma or ocular surgery showed a deep-seated intracorneal cyst in her left eye (8 o’clock corneoscleral area, dissecting into the pre-Descemet cornea). The cyst was threatening the visual axis. An epithelial cyst was diagnosed after drainage on the basis of the cyst contents. We irrigated inside the cyst using 10% trichloroacetic acid (TCA), distilled water, and 1% 5-fluorouracil (5-FU) solutions for chemical cyto-destruction of the lining epithelial cells of the cystic wall. We used a portable FD-OCT during operation to guide this procedure, without perforating the Descemet’s membrane and endothelial layer. Recurrence could be prevented after removal of the cystic tissue located in the sclera area outside of the limbus. No recurrence was noted during the 4-year follow-up.

**Conclusion:**

When treating centrally deep-seated intracorneal epithelial cysts, clinicians must consider recurrence, endothelial damage, and visual outcome. Herein we report the case of a deep-seated, intracorneoscleral epithelial cyst that was completely resolved with chemical cyto-destruction and removal of the intrascleral cystic tissue under the guidance with FD-OCT; thus, endothelial damage could be minimized.

## Background

Intracorneal cysts occasionally occur after penetrating or perforating corneal wounds. Some, however, have a congenital origin, whereby the epithelium invades during the embryonal period. These epithelial nests tend to grow and form cystic lesions that need to be treated when they threaten the visual axis.

Intrastromal cysts, which are located in the mid-stromal layer, can be treated using distilled water, 5-fluorouracil (5-FU), or 20% trichloroacetic acid (TCA) without damaging the surrounding tissues. Lamellar keratoplasty is another treatment option for the epithelial cyst removal when the cyst is not deep. A deep cyst close to the endothelium can convert to penetrating keratoplasty, however, due to perforation during lamellar keratoplasty [1].

Fourier-domain optical coherence tomography (FD-OCT) allows for excellent visualization of the cornea. Most FD-OCT systems require that patient is seated in front of the system, however, recently, new portable models enabled the patients to be examined in the supine position.

Herein, we report the case of 4 year-old girl with a growing, intractable, pre-Descemet intracorneal epithelial cyst that was treated by injecting several chemicals into the cystic cavity during the surgery under the guidance of portable FD-OCT. Corneoscleral cystectomy followed by lamellar corneoscleral transplantation was performed also to remove the cyst completely.

## Case presentation

**Fig. 1:**
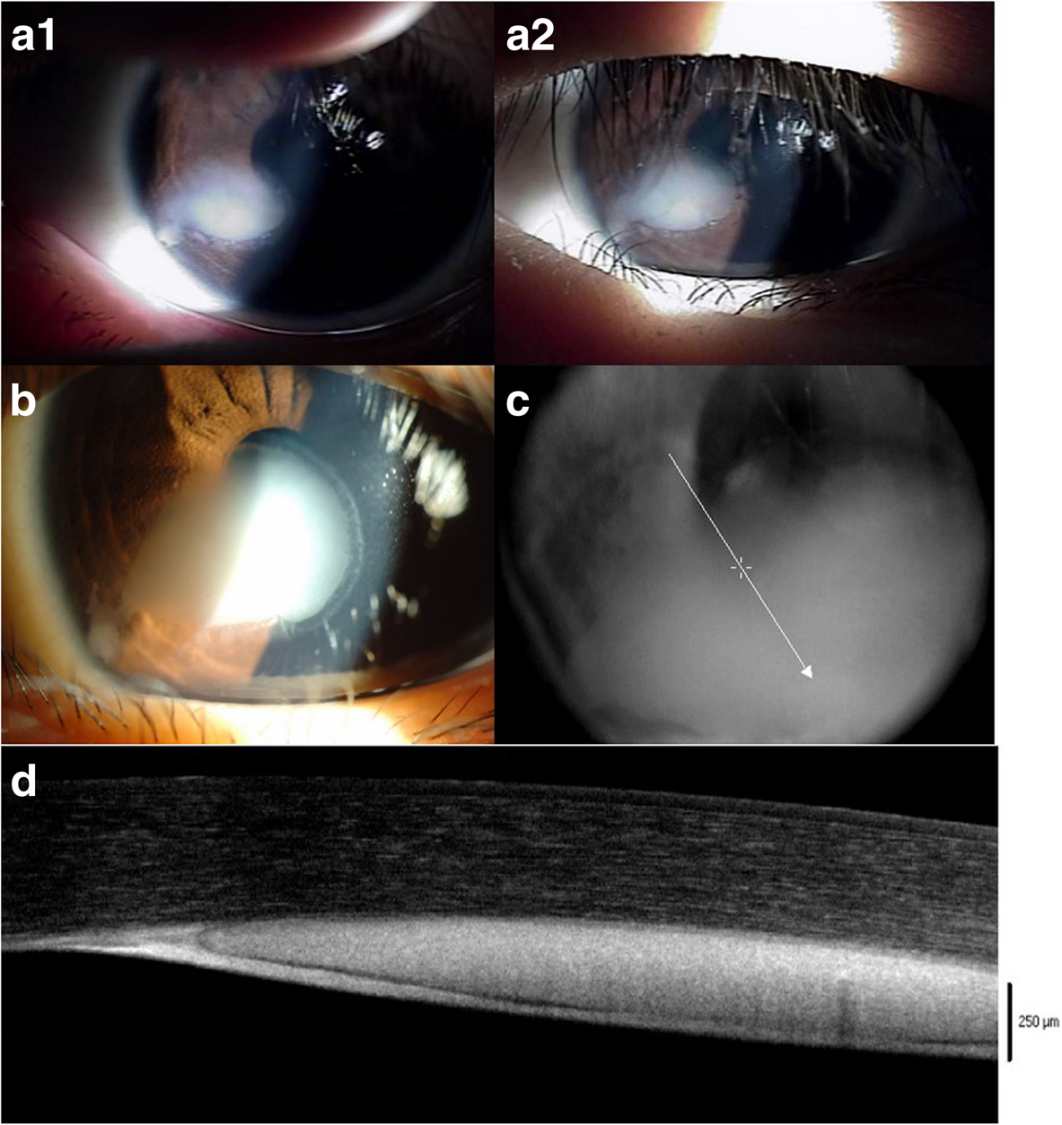
**a1**,**2** A serial photographs of the corneal lesion taken at local clinic each at 17 months and 12 months before the patient’s first visit to our clinic. **b** An enlarged corneal lesion covering the pupil-captured at the patient’s first visit to our clinic. **c** & **d** FD-OCT images of 1B, clearly showing the cystic lesion just in front of Descemet’s membrane

A 4 year-old girl was referred to the Department of Ophthalmology, Severance Hospital because of a white spot in the cornea and non-correctable vision in her left eye. Serial photographs taken before in other clinics as well as ours showed that the lesion had spread toward the visual axis (Fig. 1a1, a2, b). She had no history of trauma or surgery in the left eye. Her best spectacle-corrected visual acuity (BSCVA) was 1.0 in the right eye and 0.6 in the left eye. Slit-lamp microscopy examination revealed a whitish intrastromal mass extending from the inferonasal limbus to the para-central cornea (Fig. 1b) at the level of the pre-Descemet’s membrane layer. The ocular examination was otherwise unremarkable. Corneal FD-OCT (RTvue-100®, Optovue Inc., Fremont, CA) revealed homogenous, whitish 250 μm thick mass occupying the pre-Descemet’s membrane space (Figs. 1c & d).

**Fig. 2:**
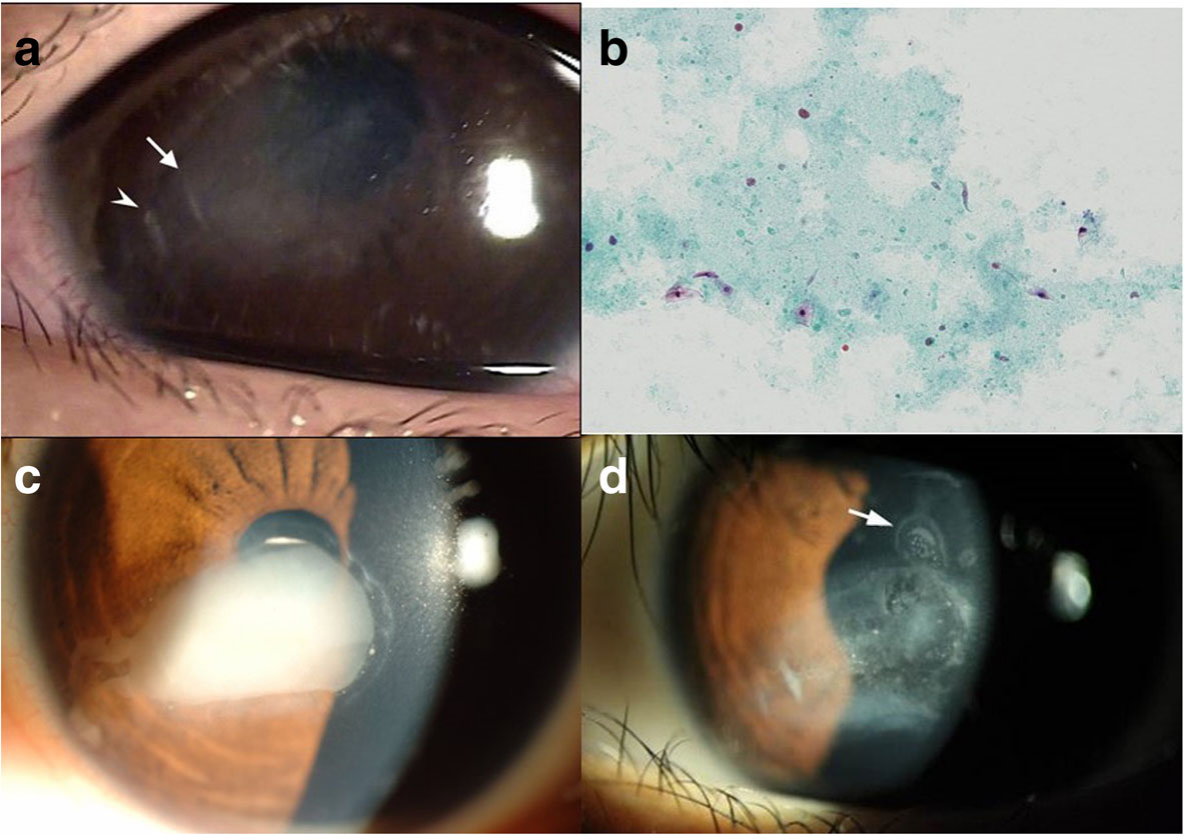
**a** During the operation, a turbid whitish cystic fluid was naturally drained from a simple incision site in the 8 o’clock cornea. **b** Histopathologic stain (Giemsa) of the exudate from the corneal lesion revealed mucin, a few stratified squamous epithelial cells, and some necrotic debris. **c** Fourteen months after surgery, the recurred corneal lesion almost covered the pupil entirely. **d** One month after the second surgery, i.e. cauterization with 2.5% TCA for 5 min, recurrence and newly formed epithelial budding of the cyst can be seen at its superior margin, extending to 12 o’clock (*arrow*). This prompted a third surgery

We performed an incision and drainage of the cyst under general anesthesia to determine the type of the cyst. Based on depth measurement data from the surface of the cornea to the anterior cystic wall by corneal FD-OCT, two parallel incisions were made in the paracentral cornea at the 8 o’clock position. The first incision was made 650 μm deep 2 mm from the limbus (arrowhead, Fig. 2a) and did not yield any drainage material. A second incision, made more centrally at a depth of 700 μm (arrow, Fig. 2a) yielded drainage of a milky, white liquid. After drainage, visual acuity in the left eye improved to 0.9 on postoperative day 1. The exudate, which was smeared on a glass slide and sent for histopathologic evaluation, revealed mucinous material and several attenuated, stratified squamous epithelial cells (Fig. 2b), resulting in a diagnosis of the intrastromal epithelial inclusion cyst.

One week after the first surgery, a thin cystic fluid was observed, and gradual development of corneal opacity was noted over the next 5 months. The patient’s BSCVA decreased to 0.8, but her left pupil was still unaffected. Fourteen months after the first operation, the corneal lesion covered most of the pupil, and the patient’s BSCVA had decreased to 0.6 (Fig. 2c).

The recurrence demonstrated the need for an additional surgery. Under general anaesthesia, we attempted chemical cauterization with TCA, as previously reported [2]. Using a Sinskey hook, we reopened the previous incision site at the 8 o’clock position to drain the cystic fluid. Following natural drainage from the previous incision site, we filled the cyst with distilled water to destroy the epithelial cell lining and attempted to make two additional incisions as evacuation outlets at the paracentral cornea at the 12 and 2 o’clock positions. Since the posterior cystic wall was very close to the corneal endothelium, we irrigated the cyst again with 2.5% TCA to attempt to destroy the epithelial cells while protecting the endothelium from damage; previous investigators had used a 20% TCA solution to treat mid-stromal cysts [2]. Irrigation with the 2.5% solution whitened the cystic wall, and the procedure was discontinued after 5 min. One week after surgery, the corneal cystic lesion had completely collapsed, and no fluid was observed.

**Fig. 3:**
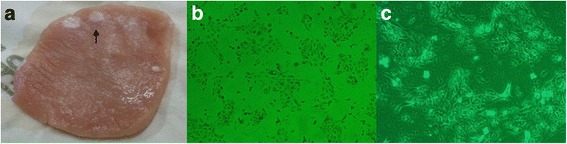
**a** Burns were made on pork tissue using 20%, 10%, 5% and 2.5% TCA. At least 10% TCA was needed to burn the tissue (*arrow*). **b** In vitro epithelial cells were treated using 10% TCA for 10 min. All the epithelium had been destroyed. **c** 60 min exposure makes the cell completely destroyed with 10% TCA

The deep-seated, intrastromal epithelial nests of the corneal lesion started to recur 2 weeks after surgery, and spreading of the nests was detected 1 month after surgery (Fig. 2d). We concluded that a 2.5% TCA solution was not strong enough to cauterize all epithelial cells lining the cystic wall. Therefore, we tested the effects of increasing concentrations of TCA in vitro in pork muscle tissue (Fig. 3a) and cultured human epithelial cells (Fig. 3b & c). A 10% solution of TCA was selected for the next irrigation trial as it was the lowest concentration that destroyed all epithelial cells and left a strong cauterization mark on the pork tissue.

**Fig. 4:**
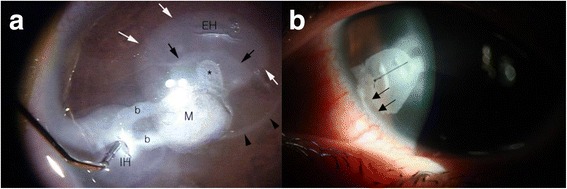
Operation findings of third operation. **a** Irrigation solutions were introduced through the main incision hole (IH) during the operation. The space which was initially filled and expanded by distilled water was indicated by the *black arrowheads*. Inside of this margin, the trace of 10% TCA made during the evacuation of the first trial of TCA can be seen as faint shaded area (*white arrows*); the evacuation hole (EH), which allows for the exit of chemicals, can be seen. From the main incision hole, two branches (b) can be seen leading to the main cyst (M), with its newly formed epithelial bud (*). Surrounding the epithelial bud, the advancing margin (*black arrows*) of the 10% TCA after second application can be seen; the margin is whiter due to the more recent cauterization. **b** Postoperative day 1: whitening of the surface epithelial layer denotes cauterization; as such, we assume that the epithelium lining the cyst was also cauterized. Deep tracts can be observed near to the limbus (*arrows*); they seem to have their origin in the limbus (*arrows*). Because the cyst wall was in contact with the chemicals, the corneal lesion, including its limbal origin, has been completely cauterized

One month after the second surgery, the cyst was irrigated with distilled water for 5 min followed by 1% 5-fluorouracil (5-FU) for 5 min to completely destroy the epithelial cells. Then, irrigation with 10% TCA for 2 min was performed twice (Fig. 4a). Even we confirmed that 10% TCA alone would be sufficient to cauterize all epithelial cells in experiment, we expected absolute removal of whole epithelial layers lining the cystic wall by adding distilled water and 5-FU irrigation. We applied one suture to the incision site and removed it 1 month after surgery (Fig. 4b).

**Fig. 5:**
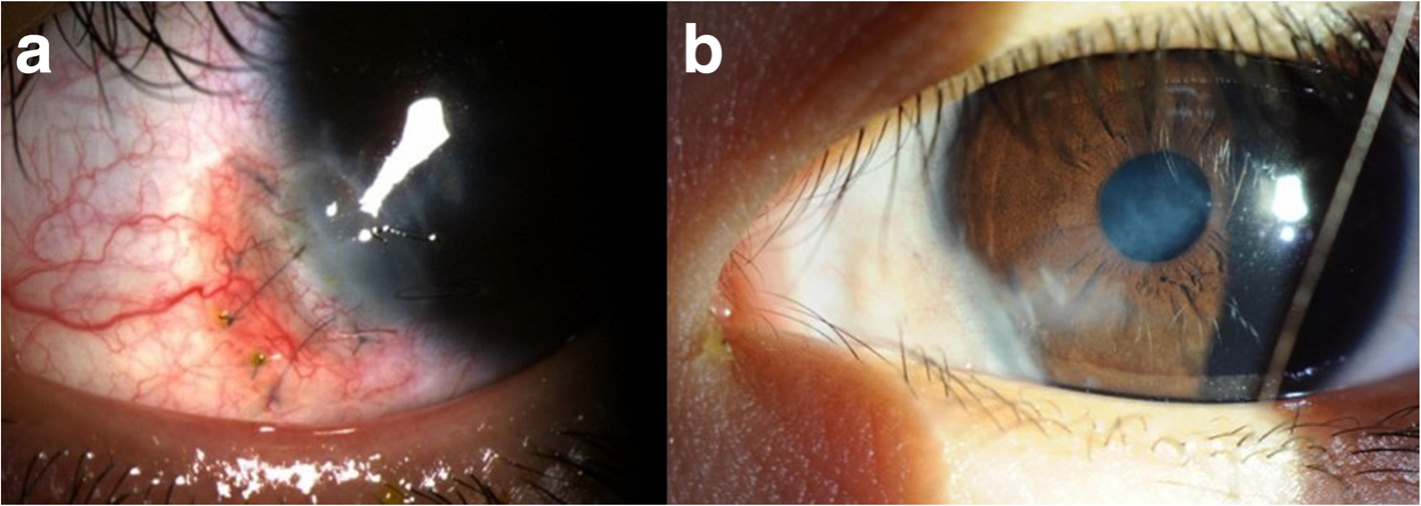
**a** One month after fourth surgery, the donated corneal tissue had adapted well; no signs of rejection sign were observed. **b** 4 years after fourth surgery, no signs of recurrence were noted

Visual acuity in the left eye improved to 0.9 after the third surgery, and recurrence was not observed for 10 months. Eleven months after surgery, the interface showed signs of recurrence, and OCT images revealed fluid collection. Evaluation of results from the previous surgery made us suspect that the original cyst was located in the sclera (arrows, Fig. 4b). Ultimately, we decided to perform a lamellar keratosclerectomy (fourth surgery) on the suspected limbal area to eliminate the epithelial nest. Based on limbal thickness measurements obtained from portable FD-OCT imaging, we dissected the sclera outside of the limbus at the 8 o’clock position under a 650 μm thick limbal-based, rectangular scleral flap and found the suspected epithelial cyst opening. Through the opening, we infused 10% TCA; we immediately observed the whitish burn tract connected to the corneal cystic lesion and confirmed that the opening was the original nest of the epithelial cyst. A 2 mm × 2 mm area of sclera, including limbus, was excised, and the lesion was replaced with donated corneal tissue using an interrupted stitch with 10-0 nylon sutures (Fig. 5a).

**Fig. 6:**
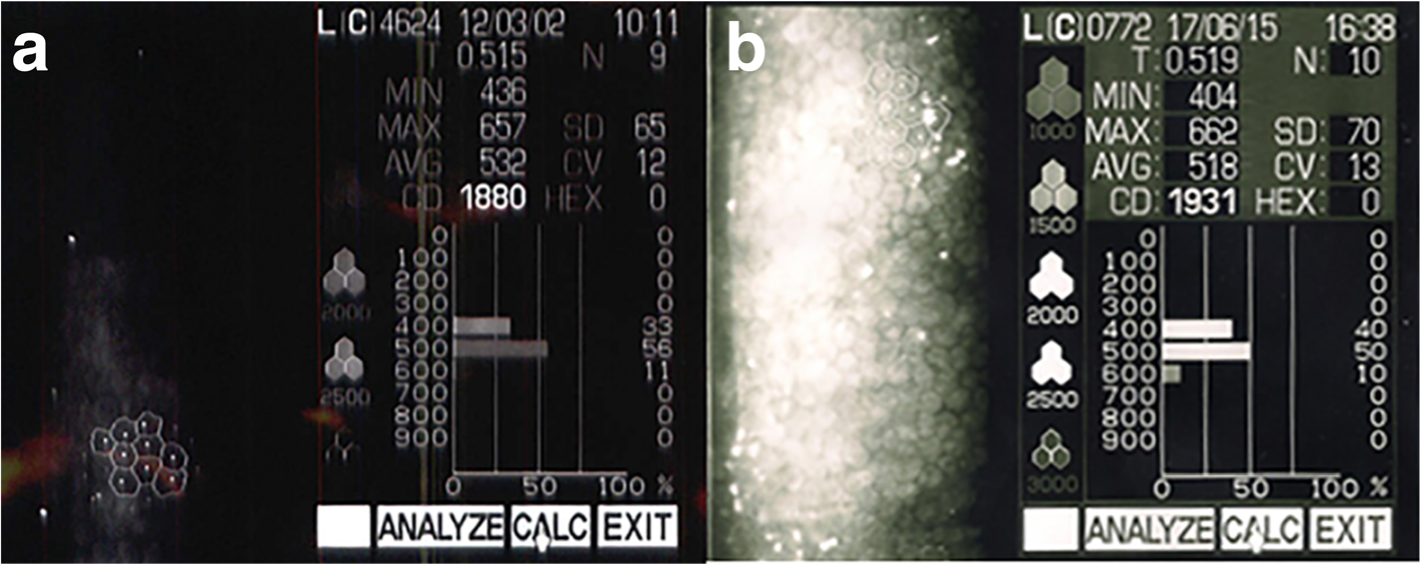
**a** Specular microscopic findings on 6 months after second surgery. Endothelial cells around central cornea where chemical cauterization had been done were counted. **b** 4 years after last surgery, endothelial cells count on central cornea remained unchanged

**Fig. 7:**
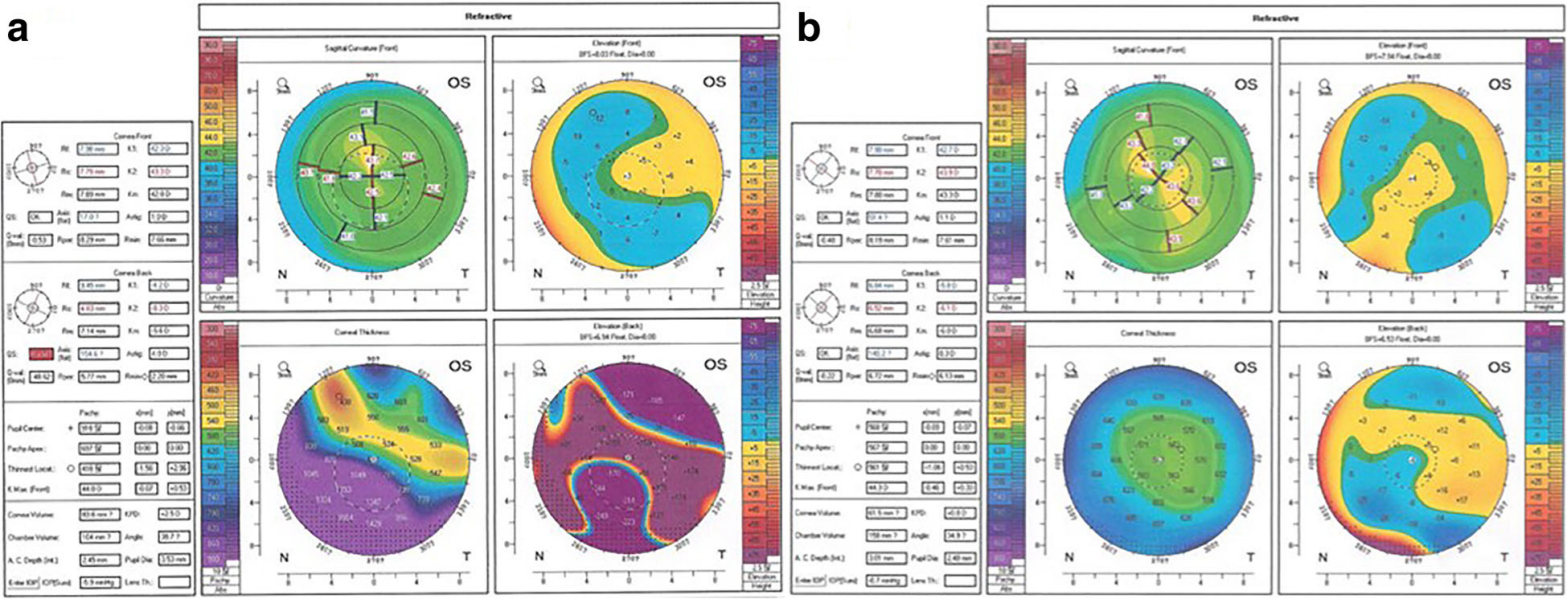
**a** Corneal topography on 9 months after first surgery. Due to recurrence of deep-seated lesion, severe posterior elevation in inferonasal area was seen, while anterior curvature map showed quite normal. **b** 4 years after last surgery, changes in anterior corneal curvature were minimal

The lesion did not recur for 4 years after the fourth surgery, and the patient’s BSCVA value was maintained at 0.8 (Fig. 5b). Even central corneal endothelial cells count after second surgery showed a significant decrease compared to right eye, it has remained unchanged for 4 years after last surgery (Fig. 6). Topographic changes were also checked before and after surgeries, which were minimal except posterior curvature (Fig. 7). Deep-seated lesion seemed not to affect anterior corneal contour.

## Discussion and conclusion

Intrastromal corneal epithelial cysts are usually ovular or circular in shape, and, in advanced stages, they generally contain an opalescent fluid [3]. In the present study, progressive enlargement of the cyst prompted surgery, which included evacuation of the fluid, chemical cauterization, and complete removal of the epithelial nest in the limbus.

The sequestration of epithelial cells in the corneal stroma, as well as their slow subsequent proliferation, likely accounted for the growth of the cyst. This abnormal positioning of the epithelial cells could have originated from trauma or developmentally, although this is still unclear [2, 4–7]. Nonetheless, our patient was young and did not have a history of ocular surgery or trauma. An ocular scar was not detected by slit-lamp microscopy or FD-OCT examinations. Therefore, we suspect that the cyst had a congenital origin, with subsequent growth and enlargement into the corneal lamella. However, minor, undetected trauma cannot be ruled out, especially if caused by a sharp object, such as a pin or stick.

In previous studies, epithelial cysts have been treated using a number of surgical procedures, with varying degrees of success [8]. When a diagnosis was confirmed, simple drainage would not be recommended because of the possibility of recurrence [9]. When the cyst has involved the visual axis, several investigators have advocated for extensive lamellar or penetrating keratoplasty [10, 11]. However, unless the entire cyst is removed, remnants lined with living cells remain at the border between the donor and virgin corneas after keratoplasty. In such cases, recurrence involving the virgin cornea might be plausible. When deep lesions are involved, any tiny perforation might induce the epithelial lining of the cyst to migrate into the anterior chamber [12]. For these reasons, it is crucial that surgeons completely eliminate cystic epithelial cells before performing lamellar or penetrating keratoplasty if they exist at the border between donor and virgin corneas.

Usually, TCA is used to destroy the epithelial cells lining the intrastromal cyst. TCA, one of the most popular chemical peeling agents, is used at concentrations varying from 15 to 50%, with higher concentrations producing deeper peels. There are reports on TCA related ocular chemical injuries [13, 14]. Since TCA has no systemic toxicity and the response to the agent is minimal or non-existent, TCA related ocular complications are usually surface limited chemical burns. As the severity of chemical ocular injury is related to the surface of contact in chemical exposure and the degree of penetration, the potential ocular complications of TCA like epithelial defects, corneal scarring and chemical uveitis, could be minimized by careful manipulation and thorough irrigation. Chemical cauterization using 20% TCA is known to prevent epithelial survival, although it can lead to scar formation [1]. Since we did not know what was a safe concentration of TCA for preserving endothelial cells that are located very close to the cystic wall, we initially applied low-strength (2.5%) TCA. Since TCA at this concentration whitened the mucinous cystic wall, a stronger solution was not needed. However, since the cyst recurred rapidly, we concluded that 2.5% TCA was not strong enough to kill all of the epithelial cells. Cauterization results from pork tissue and cultured epithelial cells with serial concentrations of TCA led us to use a 10% TCA solution. We do not know whether 10% TCA is appropriate for every corneal cyst case. However, when combined with distilled water and a 1% 5-FU solution, this was the proper concentration for killing cystic epithelial cells and protecting the nearby corneal endothelium. Whenever a solution is injected into the cystic lesion, the injection force may expand the cystic space and prompt the recurring epithelium to invade the newly created space. This expansion may be a factor in bud formation (white arrow, Fig. 2d; *, Fig. 4a) providing a weak space at the cystic margin. Allowing for evacuation of the fluid during the injection by providing a larger evacuation hole would be helpful in minimizing pressure to the cystic wall and preventing further expansion of the space.

In our case, recurrence was finally controlled after destruction of the epithelial nest by lamellar keratosclerectomy with chemical cauterization. This approach could prevent the need for more aggressive procedures, such as lamellar or penetrating keratoplasty.

In conclusion, we were able to treat the patient with several surgeries under general anaesthesia through guidance from a portable FD-OCT instrument, which allowed us to evaluate the depth of the corneal cyst. A solution of 10% TCA with distilled water and 1% 5-FU was effective in destroying the epithelial cells that lined the cyst without seriously damaging the corneal endothelial cells separated by Descemet’s membrane. Recurrence can be prevented after removal of the cyst that is located in the sclera outside of the limbus.

## Abbreviations

5-FU: 5-Fluorouracil
FD-OCT: Fourier-domain optical coherence tomography
TCA: Trichloroacetic acid
